# Epileptogenic potential of mefloquine chemoprophylaxis: a pathogenic hypothesis

**DOI:** 10.1186/1475-2875-8-188

**Published:** 2009-08-05

**Authors:** Remington L Nevin

**Affiliations:** 1 United States Africa Command, Combined Joint Task Force Horn of Africa, Camp Lemonier, FPO AE 09363, Republic of Djibouti

## Abstract

**Background:**

Mefloquine has historically been considered safe and well-tolerated for long-term malaria chemoprophylaxis, but prescribing it requires careful attention in order to rule out contraindications to its use. Contraindications include a history of certain neurological conditions that might increase the risk of seizure and other adverse events. The precise pathophysiological mechanism by which mefloquine might predispose those with such a history to seizure remains unclear.

**Presentation of the hypothesis:**

Studies have demonstrated that mefloquine at doses consistent with chemoprophylaxis accumulates at high levels in brain tissue, which results in altered neuronal calcium homeostasis, altered gap-junction functioning, and contributes to neuronal cell death. This paper reviews the scientific evidence associating mefloquine with alterations in neuronal function, and it suggests the novel hypothesis that among those with the prevalent EPM1 mutation, inherited and mefloquine-induced impairments in neuronal physiologic safeguards might increase risk of GABAergic seizure during mefloquine chemoprophylaxis.

**Testing and implications of the hypothesis:**

Consistent with case reports of tonic-clonic seizures occurring during mefloquine chemoprophylaxis among those with family histories of epilepsy, it is proposed here that a new contraindication to mefloquine use be recognized for people with EPM1 mutation and for those with a personal history of myoclonus or ataxia, or a family history of degenerative neurologic disorder consistent with EPM1. Recommendations and directions for future research are presented.

## Background

Mefloquine (Lariam^®^) is a commonly prescribed anti-malarial. Although historically the long-term use of mefloquine for malaria chemoprophylaxis has been considered safe and well-tolerated [1,2], careful prescribing is needed to minimize the potential for severe neurological adverse events, including myoclonus and seizure, for which individuals with certain neurological histories appear to be at highest risk [3]. In particular, case-report [4], case-series [5], and retrospective cohort [6,7] studies define a well-characterized pattern of increased susceptibility to seizure and other movement disorders including nystagmus and ataxia [4] with use of mefloquine among those with a personal [4-6] or family history [7] of such conditions. Additional case-reports describe, in the absence of personal or family history, the occurrence of multifocal myoclonus [8], convulsions, and seizures following both therapeutic [9] and prophylactic doses [10-12] when mefloquine is co-administered with other medications such as fluroquinolones [13], or occasionally when mefloquine administration is preceded by non-specific neurologic prodrome [14].

The U.S. package insert cautions that mefloquine *"should not be prescribed for prophylaxis in patients with active depression, a recent history of depression, generalized anxiety disorder, psychosis, or schizophrenia or other major psychiatric disorders, or with a history of convulsions" *[15]. The prevalence of these contraindications is approximately 9–10% in both military [16] and civilian [17] populations that present for malaria chemoprophylaxis, which underscores that clinicians must exercise significant care prior to prescribing and dispensing mefloquine.

A growing body of recent evidence from *in vitro *and *in vivo *studies in animal models characterize the direct neurotoxic effects of mefloquine [18-22], the ability of mefloquine to disrupt normal neuronal calcium homeostasis [21,23,24], as well as cellular calcium homeostasis [25], and mefloquine's inhibition of normal gap-junction function [26-28]. Many of these results are readily demonstrated at concentrations consistent with chemoprophylaxis [21,22,26]. Despite these advances in understanding, the precise pathophysiological mechanism remains unclear by which mefloquine predisposes to seizure people with a personal or family history of certain neurological conditions [3].

A satisfying explanation for the pathogenic mechanism of mefloquine-induced epileptogenesis would integrate current knowledge of mefloquine neurotoxicity with an understanding of the pathogenic mechanism underlying clinical conditions that mirror the clinical presentation of mefloquine-associated seizure. Furthermore, such an explanation would elucidate why these reactions occur in specific populations. This opinion paper reviews recent research and presents a novel hypothesis, consistent with these axioms, that offers an explanation for why mefloquine may predispose to seizure certain individuals with EPM1 genotype.

## Presentation of the hypothesis

### Mefloquine accumulates at high concentrations in brain tissue

Mefloquine is a highly lipotropic drug which readily crosses the blood-brain barrier [29,30] and whose efflux from neuronal tissues is mediated by the efflux pump P-glycoprotein (P-gp) [29,31]. In rat models a neuronal accumulation of enantiomeric mefloquine supports a stereoselective active neuronal efflux, and in *in vivo *mouse models, the neuronal concentration of quinine is raised in the presence of mefloquine [32]. Mefloquine is, therefore, both a substrate and an inhibitor of P-gp [31,33].

Under conditions of long-term chemoprophylaxis, mefloquine levels in plasma have been reported between 1.5 and 3.3 μM [34], but relatively few post-mortem studies have defined concentrations in brain tissue under conditions of chemoprophylaxis. One post-mortem case-series identified mefloquine in brain tissue at concentrations between 8.7 and 14 mg/kg [35], corresponding to significantly higher molar concentrations of between 21 and 34 μM. Another post-mortem study demonstrated ratios of brain white matter to serum mefloquine of 22.9:1 and 55.5:1 for (-) and (+) enantiomeric mefloquine, respectively [30]. This same study found grey matter concentrations were approximately 69% of that found in white matter [30]. Based on these data it is reasonable to conclude that mefloquine typically accumulates in brain tissue at concentrations approximately 10–30 times higher than found in serum [21].

The MDR1 gene codes for P-gp. Research postulates that polymorphisms in the MDR1 gene that result in a reduced expression of P-gp will result in lower mefloquine efflux from the brain and lead to higher brain tissue concentrations [33] than in individuals lacking these polymorphisms. Studies have demonstrated that the neuropsychiatric effects of mefloquine, as identified by earlier cohort studies [36], are more common among those with MDR1 polymorphisms [33]. It is therefore plausible that common MDR1 polymorphisms, by significantly raising neuronal levels of mefloquine, might mediate the propensity towards dose-dependent adverse events.

### Mefloquine interferes with normal gap junction functioning

Mefloquine exhibits a potent dose-dependent ability to interfere with the functioning of gap junctions, which are inter-neuronal channels created by distinct oligomeric proteins called connexins [37]. A variety of connexin (Cx) proteins have been identified and characterized, identified by their molecular weight [37]. Of these, Cx36 and Cx43 are widely distributed in neuronal tissue and show sensitivity to mefloquine.

Gap junctions formed by Cx36 predominate in neurons [38] in rat models, and play a critical role in the direct transport of ions between adjacent neurons, a process known as neuronal coupling [39]. Cx36 is found at high concentrations within the inhibitory GABAergic interneurons of the cortical striatum [39]. In mouse and rat models, Cx36 is also found throughout the ventral tegmental area [40], the neocortex [26], thalamic reticular nucleus [26], inferior olive [38,41], and possibly hippocampus [26].

Lack of functioning Cx36 has been implicated in epileptogenesis [42], although the underlying mechanism is not clear. In mice lacking Cx36, deficits are found in synchronous firing within cortical, thalamic, and brainstem circuits [26]. Gene-transfer research demonstrates inactivation of Cx36 in mature rat models results in measurable degradation in fine temporal coordination of muscle firing [41]. Mefloquine blockade of Cx36 also suppresses essential tremor when induced in mouse models [28]. At high concentrations, mefloquine also decreases the amplitude of Cx36-mediated cortical spreading depression in rat models in vitro [27].

Significant physiological effects due to mefloquine are noted at concentrations consistent with chemoprophylaxis. At concentrations of 25 μM, mefloquine inhibition of Cx36 in *in vivo *mouse models is associated with a decrease in GABAergic spontaneous synaptic currents to motor neurons [39]. In *in vitro *studies, mefloquine at concentrations as low as 10 μM significantly reduced cortical coupling, and at 25 μM mefloquine caused increases in spontaneous chemical synaptic activity [26]. In *in invo *studies in rats, mefloquine at concentrations of 25 μM antagonized the stimulatory action of modafinil, likely through decreased gap junction-mediated electrical coupling [43].

The predominant connexin expressed in neuronal glial tissue is Cx43, which is thought to permit the transfer of small molecular weight molecules critical to maintenance of cellular homeostasis [37]. Functioning gap junctions in the astrocyte network serves to dilute substances cleared from the extracellular environment, including free calcium [37]. At levels of 30 μM, mefloquine strongly inhibits Cx43 [26].

### Mefloquine disrupts intracellular calcium homeostasis

Mefloquine *in vitro *also strongly interferes with neuronal calcium homeostasis, likely through inhibitory action on the endoplasmic reticulum (ER) calcium ATPase, leading to the intracellular release of the ER calcium stores [21]. These effects have been consistently noted in rat neurons *in vitro *at concentrations of between 10–30 μM [21,22]. In a dog model, mefloquine inhibits inositol-1,4,5-phosphate (IP3)-mediated calcium release from neuronal microsome stores, although at slightly higher molar concentrations [24], suggesting a role for mefloquine in IP3-receptor (IP3-R)-mediated signal transduction processes.

### Mefloquine produces dose-dependent neuronal toxicity

In rat model experiments, mefloquine produces permanent and dose-dependent brain stem lesions in the nucleus gracilis, nuclear cuneatus, and solitary tract [20]. These lesions were accompanied by dose-dependent impairments in motor performance consistent with loss of vestibular or nucleus gracilis-dependent proprioceptive function. Inhibition of cytoplasmic calcium homeostasis might trigger a shutdown of protein synthesis [23], resulting in an induction of proapoptotic mediators and contributing to the drug's neurotoxic effects [21]. Such lesions were moderately demonstrated at doses of 187 mg/kg, corresponding with rat plasma concentrations of 2.1 μg/mL [20] or approximately 5.6 μM plasma concentration.

### Significance of EPM1 mutation

Unverricht-Lundborg Disease (ULD) is an inheritable, autosomal recessive, degenerative form of epilepsy characterized as progressive and myoclonic, type 1 (EPM1) [44]. ULD is generally diagnosed prior to age 18, and in half of cases the first presentation is tonic-clonic seizure [44]. EPM1 homozygosity is characterized by the early onset of progressive ataxia, incoordination, intentional tremor and dysarthria.

Genetic studies have revealed that EPM1 is associated with loss of function in the cystatin B gene (CSTB) in over 90% of cases [45] due to a dodecamer repeat expansion in the CSTB promoter [45] that results in significant downregulation of mRNA expression. CSTB protects against the proteolytic activity of cathepsin cystein proteases, particularly capthepsin B [45], and the main function of cytosolic CSTB in normal physiology is thought to be protection from lysosomal protease leakage [46]. However, in CSTB knock-out mice, ataxia, myoclonic seizures, rapid cerebellar degeneration and apoptosis are noted [47] and are associated with significant increases in transcription of cathepsin S and markers of glial activation [48], themselves consistent with reactive changes suggestive of widespread cortical and subcortical grey matter damage [49]. CSTB knock-out mice also demonstrate increased susceptibility *in vivo *to severe seizure, including generalized tonic-clonic seizures, and *in vitro *exhibit neuronal hyperexcitability characterized by decreased seizure threshold [50].

Additionally, mouse studies demonstrate that EPM1 heterozygosity results in lower cystatin B expression than wild-type mice and is associated also with near-universal mild neurogeneration and neuronal atrophy [51]. In some cases heterozygosity also might be associated with mild signs and symptoms of ULD, including ataxia, motor incoordination and myoclonus [51].

These results suggests that CSTB deficiency creates impairments in the normal spectrum of neuronal physiologic safeguards, which predisposes EPM1 heterozygotes to neuronal hyperexcitability and hastens neuronal cell death with seizure [50].

### Epileptogenic potential of mefloquine in EPM1 heterozygotes

Despite the absence in the literature of such events being reported, and based solely on biologic plausibility, the previous findings support the novel hypothesis that EPM1 heterozygosity might substantially increase the risk of seizure among subjects exposed to mefloquine, particularly in the presence of an MDR1 polymorphism or other condition, including the co-administration of a P-gp inhibitory drug that raises brain concentrations of mefloquine.

Among people with EPM1 mutation and exposed to mefloquine at an as-yet undetermined threshold grey-matter concentration, inherited predisposition to and reductions in physiologic protection from apoptosis are postulated to be exacerbated by a mefloquine-induced dysfunction among a spectrum of ordinarily complementary neuronal physiologic safeguards that become altered in mefloquine's presence. These altered physiologic safeguards include a loss of normal neuronal calcium homeostasis via mefloquine-induced release of ER calcium stores, altered glial cell function via mefloquine-induced Cx43-mediated gap junction dysfunction; and decreased GABAergic cortical inhibition and coupling and increased motor-neuron synaptic activity via mefloquine-induced Cx36-mediated gap junction dysfunction.

Although the precise role of altered astrocyte gap function in mediating apoptosis is not yet clear [52], it might be that in the presence of mefloquine-induced Cx43 dysfunction, increased neuronal cellular calcium resulting from inhibition of ER calcium ATPase is mediated less effectively by the astrocyte network, which then more readily induces neuronal proapoptotic mechanisms themselves promoted by mefloquine.

Simultaneously, it might be that mefloquine-induced Cx36 dysfunction results in decreased inhibitory GABAergic input and increased spontaneous synaptic activity. Supporting this postulate are results from *in vivo *mouse models, which demonstrate that high-dose mefloquine induces seizure, and that this effect is blocked by potent GABA agonists [53].

Given the simultaneous loss of multiple neuroprotective safeguards associated with mefloquine administration, it is, therefore, plausible that the first manifestation of seizure in EPM1 heterozygotes might occur with mefloquine prophylaxis.

## Implications and testing of the hypothesis

Consistent with case reports of tonic-clonic seizures occurring during mefloquine chemoprophylaxis among those with family histories of epilepsy [7], out of an abundance of caution and based on biological plausibility, consideration should be given to a contraindication to mefloquine use among those with EPM1 mutation; or absent formal genetic diagnosis, among those with a personal or family history of seizure, myoclonus, ataxia, or degenerative neurologic disorder consistent with EPM1. Such a contraindication would expand upon and clarify existing U.S. package-insert guidance that limits existing neurologic contraindications to a personal *"history of convulsions."*

The prevalence of EPM1 homozygosity is as high as four per 100,000 in some western populations [44], which suggests that the genotypic frequency of EPM1 heterozygosity under Hardy-Weinberg equilibrium could exceed one percent. Such underlying prevalence might be consistent with the incidence of observed neurologic adverse events with mefloquine prophylaxis, particularly when coupled with an as-yet-unclear population prevalence of either MDR1 polymorphisms, co-administration of medication which may further inhibit P-gp function, or other conditions such as liver dysfunction which may decrease metabolism and, therefore, lead to increases in neuronal mefloquine concentrations.

Post-marketing studies, including genetic studies testing for EPM1 mutation among people who have suffered severe seizures following the prophylactic or therapeutic use of mefloquine should be conducted to further elucidate this alleged risk. Additionally, post-mortem studies should be conducted also to better characterize the correlations between brain concentrations of mefloquine, prevalent genetic mutations including EPM1 and MDR-1, co-morbid liver dysfunction including alcohol- and drug-induced impairments, and the extent of neuronal apoptosis that occur under conditions of chemoprophylaxis.

Although obtaining access to large numbers of post-mortem specimens exposed to mefloquine chemoprophylaxis has proven challenging historically, ongoing U.S. military efforts in malarious areas, including Afghanistan, Africa, Asia, and South America, present an unfortunate but meaningful opportunity to improve understanding in this area. Because mefloquine has until recently served as primary chemoprophylaxis for U.S. service members deployed to some of these areas, [16], and may still be used by a significant proportion of the U.S. military population [54], the direct measurement of brain mefloquine concentrations [35] and the extent of neuronal apoptosis during U.S. military-directed post-mortem examination, would assist greatly in improving understanding of these and other recently postulated mechanisms of mefloquine neurotoxicity [55].

## Abbreviations

ATP: Adenosine Triphosphate; CSTB: Cystatin B; Cx: Connexin; EPM1: Progressive Myoclonic Epilepsy; Type 1; ER: Endoplasmic Reticulum; GABA: Gamma Aminobutyric Acid; IP3: Inositol-1,4,5-Phosphat; IP3-R: IP3 Receptor; μM: Micro Molar; ug/mL: Microgram per Militer; mg/kg: Miligrams per Kilogram; MDR1: Multi-Drug Resistance 1 Gene; P-gp: P-Glycoprotein; ULD:Unverricht-Lundborg Disease; U.S.: United States.

## Competing interests

The author declares that he has no competing interests.

## Disclaimer

The opinions expressed are those of the author and do not necessarily reflect those of the Department of the Army, the Combined Joint Task Force Horn of Africa, the United States Africa Command, or the Department of Defense. The author wrote this paper as part of his routine activities and no external support or funding was obtained.
